# Uterine Rupture: A Case Series From a Tertiary Care Center in Northern India

**DOI:** 10.7759/cureus.47446

**Published:** 2023-10-21

**Authors:** Sakshi Agrawal, Ankita Balara, Lalit Kumar

**Affiliations:** 1 Obstetrics and Gynecology, Institute of Medical Sciences, Banaras Hindu University, Varanasi, IND; 2 Urology, Institute of Medical Sciences, Banaras Hindu University, Varanasi, IND

**Keywords:** pregnancy, trimester, third, rupture, uterus

## Abstract

Uterine rupture is a rare, preventable catastrophic condition in obstetrics. It is associated with high fetal and maternal morbidity and mortality. Its timely diagnosis and swift management may result in better outcomes. Here, we study the presentations and outcomes of three cases of ruptured uterus in the third trimester. Three cases of uterine rupture were presented in the Department of Obstetrics & Gynaecology between the 1st of April 2022 and the 31st of March 2023 in the third trimester, at 29, 33, and 37 weeks, respectively. The mean age of the patients was 25.33 (19-29) years. All three patients had a history of scarred uterus and were diagnosed with clinical suspicion of rupture. Other symptoms were hematuria and absent fetal cardiac activity in the second case; in the third case, she had tenderness in her previous cesarean section scar. Two out of three patients required urinary bladder repair and uterus repair. All patients did well after surgery, leading to no mortality. Our case series findings suggest that the clinical presentations of ruptured uterus vary. A history of previous cesarean section, and findings suggestive of peritonitis like tachycardia, shock, pallor, and absence of an intact uterus should raise suspicion of a ruptured uterus. Searching for non-gynecological causes in such clinical presentations might delay the crucial surgical intervention that leads to mortality, morbidity, and future obstetrics function.

## Introduction

Rupture of the uterus is an uncommon and often catastrophic condition. It involves total disruption of the wall of the pregnant uterus with or without extrusion of its contents. Uterine rupture involves the entire thickness of the uterine wall, resulting in communication between the uterus and peritoneal cavities. Uterine rupture is associated with high fetal and maternal morbidity and mortality. It is a preventable condition and timely diagnosis and swift management may result in better outcomes. It may occur in approximately 0.05% of all pregnancies, 0.8% after a previous lower segment cesarean section (LSCS), and >5% after a classical cesarean section. [1,2] In a similar condition uterine scar rupture has separation of the scar along its entire length often with the involvement of the amniotic membranes. Scar dehiscence has an incidence of 0.6% in pregnancies with previous cesarean section and has a more favorable outcome for both mother and fetus than uterine rupture. [2,3]

The sequels of uterine rupture depend on the elapsed time between the diagnosis of uterine rupture and the quality of care provided. This condition can have catastrophic consequences related to mother and fetus if not managed swiftly. Maternal complications can be hemorrhage, need for multiple blood transfusions, shock, associated visceral injuries like urinary bladder, hysterectomy, sepsis, ICU admissions, maternal mortality, etc. Fetal complications can be divided into fetal and maternal consequences. Fetal consequences are fetal hypoxia/anoxia, sepsis, admission to the neonatal intensive care unit, and neonatal mortality, etc. So early diagnosis is the key to success. After diagnosis, vital measurements, quick supportive and resuscitative therapy like intravenous fluid, blood transfusion, etc. should be started [2,4]. Along with this, all preparation for urgent surgery and transfer to the operation theater, operation theater preparation, and coordination with the anesthetist and other required surgical specialties should be done.

Hysterectomy is considered the treatment of choice in patients with multiple intractable hemorrhages or uterine rupture sites. Repair with or without bilateral tube ligation is done in young and stable patients. Some patients may even require assisted urinary bladder repair and ligation of internal iliac arteries to control bleeding. Repeat cesarean section is done at 36 weeks of gestation in patients with previous uterine repair. The objectives of this study were to determine the incidence, risk factors, clinical presentation, complication, management, and maternal and fetal outcomes in patients with uterine rupture in pregnancy [3,4].

## Case presentation

Three cases of uterine rupture presented in the third trimester at 29, 33, and 37 weeks, respectively, in the Department of Obstetrics & Gynaecology between the 1st of April 2022 and the 31st of March 2023. The mean age was 25.33 (19-29) years. All three patients had a history of a scarred uterus. All patients were diagnosed with clinical suspicion of rupture. Other symptoms were hematuria and absent fetal cardiac activity in the second case while the third case had previous cesarean section scar tenderness. Two out of three patients required urinary bladder repair along with repair of the uterus. All patients did well after surgery, leading to no mortality. The detailed history, examination, and management findings of all three cases are described below.

First case

An unbooked, 28-year-old female G2P1L1, was referred from the district hospital as a case of a previous cesarean section with term pregnancy and acute pain in the abdomen on admission to the labor room. On examination, her vitals were stable. Her previous cesarean delivery was 10 years back for the failure of the progression of labor She has no positive history of previous medical disorders/surgeries.

On presentation, the patient’s blood pressure was 120/84 mmHg with a pulse rate of 100 bpm and urine albumin of 4+ with absent fetal cardiac activity on ultrasound. One hour after admission her vitals started deteriorating, she had tachycardia at 126 bpm, her blood pressure started falling to 90/60 mmHg, and her respiratory rate increased to 30 breaths per minute 

On examination patient looked pale, abdominal distension and rebound tenderness were present, and uterine contour could not be felt. CBC was suggestive of 8gm/dl hemoglobin. A bedside doppler was showing an absent fetal heartbeat. On the suspicion of a ruptured uterus patient consented to exploratory laparotomy with all risks explained.

Intraoperative findings were suggestive of 500 cc of hemoperitoneum, the dead fetus was lying within the abdominal cavity, the fetus was delivered, and cord pulsation was absent. Uterine rupture presented 4-5 cm involving the entire lower segment transversely which also extended vertically downwards towards the cervix (3-4 cm) bladder rupture present extending transversely along the dome 3-4cm also vertically downwards along the posterior wall (2 cm).

**Figure 1:**
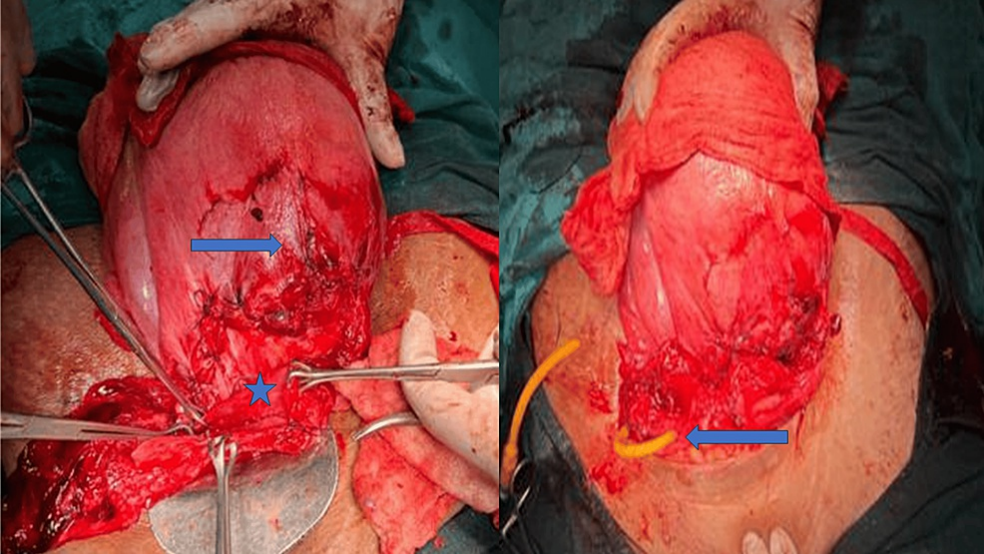
Repaired uterine rupture and bladder Left-side arrow - repaired uterus, star mark - bladder injury, right-side arrow - repaired bladder with suprapubic catheter in situ

Intra-op, a urologist was called, the bladder rupture was repaired, and a suprapubic catheter was placed. Uterine rupture was also repaired and the uterus was conserved (Figure 1). Complete hemostasis was secured. Four units of packed RBCs and two units of fresh frozen plasma (FFP) were given, and a drain was placed intraoperatively.

Postoperatively, the patient was admitted to the high-dependency unit (HDU). Complete suture removal was done on postoperative day 10. The patient was discharged on day 10 with proper follow-up advice. The per urethral and suprapubic catheters were removed two weeks and two weeks after surgery, respectively. She was counseled regarding the risk of rupture in future childbirth, and contraceptive counseling was also done.

Second case

An unbooked 29-year-old female G2P1L1 presented to triage with complaints of amenorrhoea for eight months, decreased fetal movements since 1 day, and epigastric pain for 4 hours. She was shifted to the labor room. Her last childbirth was a cesarean done for cephalopelvic disproportion (CPD). There were no previous medical disorders, no relevant family history, and no previous surgeries.

On general examination, the patient was severely pale, her pulse rate was 130 bpm, her blood pressure was 80 systolic, her respiratory rate was 32 breaths per minute, and her oxygen saturation (SpO2) was 94 on room air. The per-abdominal examination had abdominal guarding and rigidity was present, with uterine fetal bradycardia of 100 bpm fetal heart rate. On the suspicion of a ruptured uterus patient was shifted for exploratory laparotomy with all risks explained.

Intraoperative findings were suggestive of 1000 cc of hemoperitoneum, the ensac fetus was lying within the abdominal cavity with the placenta separated and attached to the lateral part of the uterus. The fetus was delivered and attended by the pediatrician, and cord pulsation was present. It was a female baby weighing 1305 gm, immediately sent to the NICU for stabilization. There was uterine rupture of 4-5 cm in the lower segment transversely, which also extended vertically upward toward the fundus (8 cm). An intraoperative urologist call was given, on examination. No evidence of bladder injury was confirmed by retrograde instillation with methylene blue dye with normal saline.

**Figure 2:**
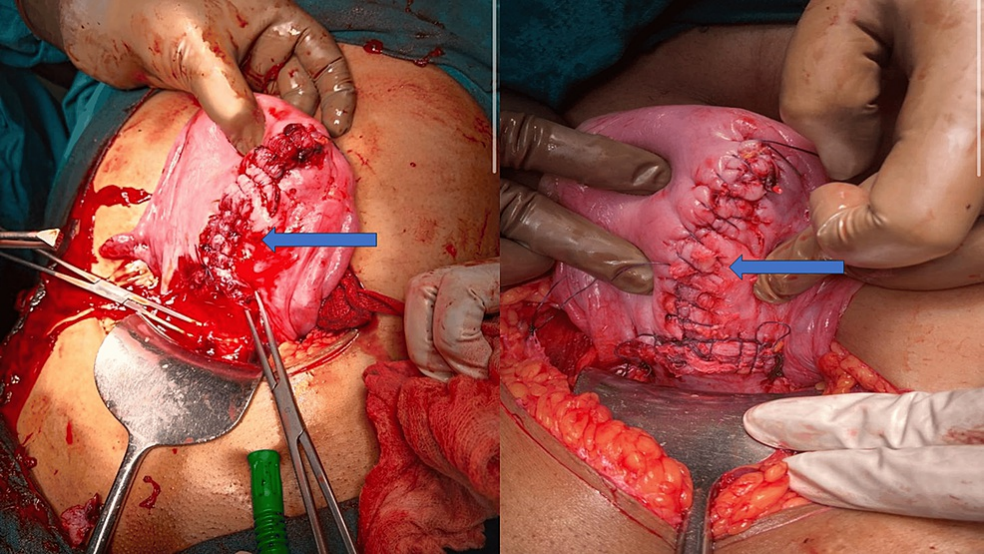
Left picture - T-shaped uterine rupture extending till the fundus; right picture - the uterus after repair

The uterine rupture was repaired and the uterus was conserved (Figure 2). The patient had atonic postpartum hemorrhage (PPH) medical therapy given, and a massive transfusion protocol was initiated along with bilateral uterine artery ligation. Five units of packed RBC and four units of FFP were given. Postoperatively, the patient was admitted to the HDU and the postoperative period was uneventful. The patient was discharged on postoperative day 7 with follow-up advice, contraceptive counseling, risk of rupture during future childbirth, and the need for proper antenatal care in the future.

Third case

An unbooked 19-year-old G2P1L1, at 29 weeks gestation, presented to triage with acute onset of pain in the abdomen for three hours. On presentation, the patient was in shock at measuring vitals, initial resuscitation was started, and she was shifted to the labor room.

**Figure 3:**
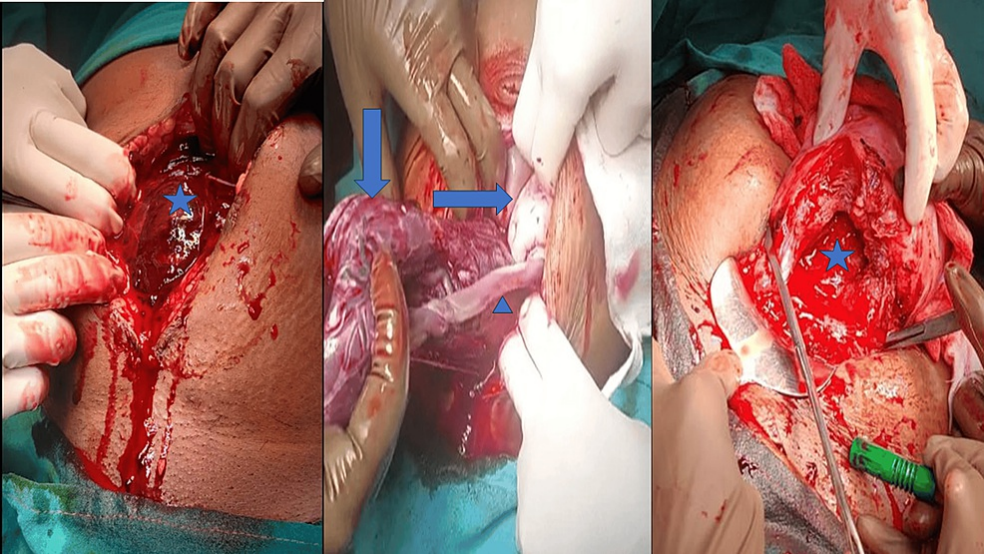
Left picture - star mark showing hemoperitoneum; middle picture - verticle arrow, horizontal arrow, and arrowhead showing placenta, fetus, and umbilical cord, respectively; right picture - star mark showing the ruptured uterus transversely

Her first cesarean delivery was performed one year ago for CPD. On presentation, the patient’s blood pressure was 70/50 mmHg with a pulse rate of 120 bpm and with absent fetal movements. On examination, the patient was severely pale, and her abdomen was distended, showing signs of peritonism, tenderness, rebound tenderness, and guarding. Uterine contour was absent. CBC showed a hemoglobin of 6 gm/dl. A bedside Doppler showed fetal bradycardia. On the suspicion of a ruptured uterus, the patient consented to exploratory laparotomy with all risks explained. Intraoperative findings were suggestive of 1000 cc of hemoperitoneum, and the ensac fetus was lying within the abdominal cavity. The fetus was delivered and cord pulsation was present. It was a female baby weighing 1186 gm, and she was immediately sent to the NICU for stabilization. There was a uterine rupture of 5-6 cm in the lower segment transversely and the bladder was high up and adhered to the ruptured site (Figure 3). An intraoperative urologist call was given. He found a serosal tear over the dome of the bladder (4 cm) transversely. This was confirmed by retrograde instillation with methylene blue dye with normal saline. A per urethral catheter (PUC) was in situ and palpable inside the bladder.

Uterine rupture was repaired and the uterus was conserved. Bilateral uterine artery ligation was done. Hemostasis secured and per op, five units of packed RBC and four units of FFP were transfused. Postoperatively, the patient was admitted to the HDU, and this period was uneventful. The patient was discharged on postoperative day 7 and recovered well. She was briefed and counseled about the risk of recurrence in future pregnancies and contraception advice was given.

## Discussion

Rupture of the gravid uterus is an unexpected, rare, and potentially life-threatening, devastating complication for both mother and fetus. It usually involves the disruption of layers of the uterus, leading to maternal and fetal sequelae. It still constitutes one of the most serious obstetrical emergencies [5]. Despite the advances in modern medicine, it continues to cause adverse fetal and maternal health consequences.

There are several predisposing risk factors for uterine rupture like a history of prior cesarean section/uterine surgeries, multiparity, obstructed labor, malpresentation, fetal macrosomia, labor dystocia, etc. Among them, the history of previous cesarean sections contributed to approximately 66% of cases of all uterine ruptures, especially in developed countries. However, in developing countries, obstructed labor and poor access to hospital delivery also contribute significantly. Therefore, one has to be cautious while managing a mother with the above-mentioned risk factors and repeat cesarean section should be considered in women with a scarred uterus. Cesarean delivery can also prevent more than 42.1% of cases of rupture of the uterus as reported by Hamilton et al. [6].

As reported in the literature, and in our case series, the most common risk factor for uterine rupture was a prior cesarean delivery. Over the last few years, the rate of cesarean section has increased, leading to more women presenting to the labor ward for delivery with a scarred uterus. This predisposes them to a higher risk of uterine rupture [7]. The risk of uterine rupture also depends on the type of prior uterine incision (low transverse, low vertical, classical, or unknown). Landon MB et al. reported uterine rupture rates of 0.5%, 0.7%, and 2.0% for unknown scars, low transverse incisions, and low vertical incisions, respectively [8]. An approximately 4% to 9% risk of uterine rupture has been found with a T-shaped or classical cesarean uterine incision [9]. So classical incisions seem to carry higher risk as compared to low transverse incisions. In our study, all patients had unknown scars. So it is challenging to plan management in patients with an unknown prior scar. In such situations, obstetricians should infer the type of uterine incision and likely the risk of uterine rupture in such a group of patients. As in our series, one patient had a prior cesarean delivery for obstructed labor, and two patients for CPD. The likely scar in our referred patient might be a classical incision predisposing to uterine rupture.

As described in the literature, uterine rupture is a catastrophic condition for both mother and fetus. Its consequences depend on the time interval between an event of uterine rupture and its surgical management. The first patient should be managed with immediate vital parameters measurement, intravenous fluid and blood transfusion, and other supportive and resuscitative measures for the management of shock along with preparation for urgent surgery after the diagnosis of uterine rupture. This helps in stabilizing the patient and reducing maternal mortality. It can be managed by repairing uterine rupture and conserving the uterus or subtotal or total hysterectomy. The type of surgery can be decided by an obstetrician based on the type, location, and length of uterine rupture, residual normal uterus, severity of hemorrhage, parity, mother’s desire for future childbearing, general condition of the patient, associated other visceral injuries, etc. There are two different schools of thought regarding treatment, Some authors consider hysterectomy as the procedure of choice; whereas others recommend surgical repair of the uterus as a viable alternative [10,11]. In our case series, successful repairs were achieved in all three patients. Such patients need to be managed carefully for further delivery in subsequent pregnancies, as the previous repair of a ruptured uterus may enhance the chances of its recurrence with an incidence of approximately 4.3-19% [12,13]. Therefore, a low threshold for repeat cesarean section should be kept for such patients.

The literature varies regarding maternal mortality in uterine rupture. It varies from 0% to 13% as described in different studies [14-16]. The mortality in our small case series came out to be 0%. It reflects the importance of early accurate diagnosis and prompt management. It also helps in reducing fetal complications like hypoxia, anoxia, acidosis, fetal mortality, etc. Holmgren C et al. found that a time interval of less than 30 minutes from diagnosis to delivery was associated with good long-term neonatal outcomes [17]. The time delay between the onset of rupture and delivery contributed to high neonatal mortality, as demonstrated also in the first case in our case series. All three patients referred to us were unbooked and came to the emergency, so urgent management is required in all cases. The primary health care centers should have quick transportation facilities for referral to higher tertiary care centers immediately. Therefore, we emphasize the importance of early identification of at-risk women for uterine rupture and early referral to a tertiary care center.

Early identification of non-reassuring fetal heart rate patterns can help the obstetrician to suspect uterine rupture early. However, there is a lack of availability of electronic fetal monitors in our institution and the majority of the institutions in our country. We presume that once continuous electronic fetal monitoring facilities are more readily available, the incidence of grave maternal and neonatal consequences can be reduced.

## Conclusions

In conclusion, uterine rupture is a major contributor to maternal morbidity and neonatal mortality. Identifying these high-risk women, early diagnosis, immediate transfer, and optimal prompt management must be overemphasized to avoid adverse feto-maternal complications. Extreme caution should be taken when managing a patient with a previous uterine scar and attempting a trial of labor. Increased accessibility to good obstetric care and prompt referral system to equipped facilities with availability of transportation services is essential for developing countries to avoid these catastrophic emergencies.
